# Tailoring diamond’s optical properties via direct femtosecond laser nanostructuring

**DOI:** 10.1038/s41598-018-32520-0

**Published:** 2018-09-24

**Authors:** M. Martínez-Calderon, J. J. Azkona, N. Casquero, A. Rodríguez, Matthias Domke, M. Gómez-Aranzadi, S. M. Olaizola, E. Granados

**Affiliations:** 1 CEIT-IK4 & Tecnun, Manuel Lardizabal 15, 20018 Donostia, San Sebastián Spain; 2 SLAC National Accelerator Laboratory, Menlo Park, CA 94025 USA; 3 Josef Ressel Center for Material Processing with Ultrashort Pulsed Lasers, Research Center for Microtechnology Vorarlberg University of Applied Sciences, Dornbirn, Austria

## Abstract

We demonstrate a rapid, accurate, and convenient method for tailoring the optical properties of diamond surfaces by employing laser induced periodic surface structuring (LIPSSs). The characteristics of the fabricated photonic surfaces were adjusted by tuning the laser wavelength, number of impinging pulses, angle of incidence and polarization state. Using Finite Difference Time Domain (FDTD) modeling, the optical transmissivity and bandwidth was calculated for each fabricated LIPSSs morphology. The highest transmission of ~99.5% was obtained in the near-IR for LIPSSs structures with aspect ratios of the order of ~0.65. The present technique enabled us to identify the main laser parameters involved in the machining process, and to control it with a high degree of accuracy in terms of structure periodicity, morphology and aspect ratio. We also demonstrate and study the conditions for fabricating spatially coherent nanostructures over large areas maintaining a high degree of nanostructure repeatability and optical performance. While our experimental demonstrations have been mainly focused on diamond anti-reflection coatings and gratings, the technique can be easily extended to other materials and applications, such as integrated photonic devices, high power diamond optics, or the construction of photonic surfaces with tailored characteristics in general.

## Introduction

**Figure 1:**
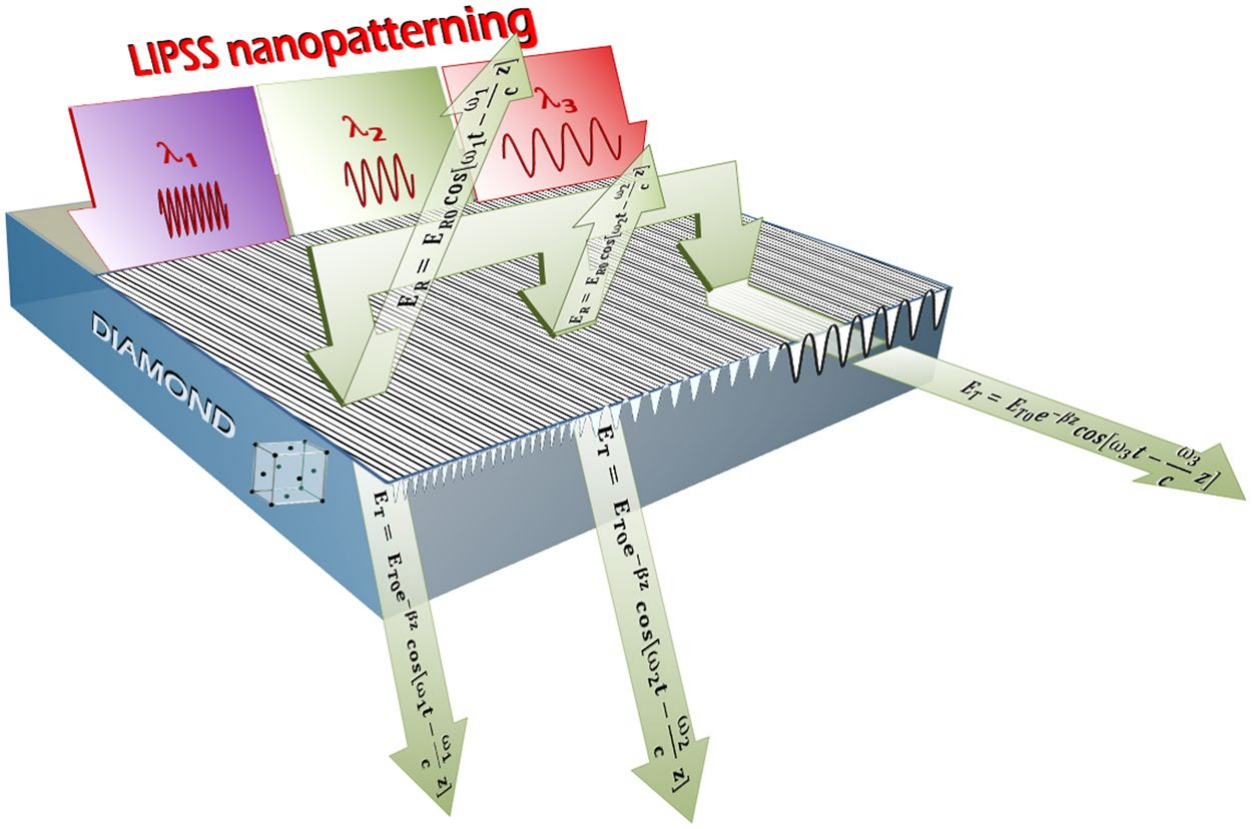
Diamond optical properties highly depend on the LIPSSs periodicity and aspect ratio.

Unlike natural diamonds, which are created by slow geological processes involving extreme pressures and temperatures, large synthetic high quality diamond wafers can be efficiently fabricated in laboratory environments^1,2^. Synthetic diamonds are chemically and biologically inert, thereby allowing them to be used in extreme chemical, physical or radioactive environments that would destroy ‘weaker’ materials. Optical and thermal applications benefit from its large optical band-gap (5.47 eV), which makes it ideal for nano-photonic and electronic devices such as high frequency FET transistors^3^, integrated frequency combs^4^ or high power switches^5,6^. Diamond’s ultra-wide spectral transparency also enables the extension of high power optical devices to new operating wavelengths, including the difficult to access deep ultraviolet region^7,8^. Its high refractive index in the UV-VIS range makes diamond one of the best candidates for the new trend of all-dielectric photonic metamaterials^9^. In addition, the precise control and application of diamond’s color centers has become crucial for quantum computing, sensing and labeling applications^10^. Most of diamond’s photonic applications benefit from 3D structuration of its surface with nanometer accuracy^10^. Figure 1 depicts a schematic example where the periodicity of the nanostructures modulates the optical transmission of diamond at a range of different wavelengths.

The development of simple and reliable techniques for the fabrication of diamond components including diffraction gratings^11^, moth-eye antireflection coatings^12^, field emitters^13,14^, waveguides, resonators and couplers for quantum processing^15^ is a very active area of research. Perhaps, the most important bottleneck for diamond surface nano-processing is that the established techniques used for patterning other semiconductors such as silicon wafers are either ineffective or require complicated fabrication steps. Common specialist nano-fabrication techniques include reactive ion etching, ion implantation with chemical etching, and laser ablation, which is usually too coarse for most optical applications^16^. Etching of diamond surfaces employing femtosecond deep-UV radiation is being currently explored with promising results, although still remains to be exploited effectively for nano-fabrication^17^.

Recently, femtosecond laser induced periodic surface structuring (LIPSSs) has proven its potential in metallic materials^18^, dielectrics^19^, polymers^20^ and thin films^21^. Compared to traditional laser ablation techniques, femtosecond laser nanomachining delivers extreme peak irradiance with minimal thermal effects, offering the possibility of surface processing in open-air atmosphere and therefore avoiding expensive vacuum technologies, while being free of chemicals and relatively fast. One of the most attractive properties of LIPSSs-based patterning is that is highly controllable, since it depends mainly on the material in use, the laser wavelength λ, the number of applied pulses per spot *N*, the angle of incidence *θ* and the polarization state of the laser electric field^22^.

In terms of nanostructure periodicity Λ, LIPSSs are divided into two main groups: the ones with Λ close to the irradiation wavelength are called low spatial frequency LIPSSs (LSFL), while structures with periods significantly smaller than λ are referred to as high spatial frequency LIPSSs (HSFL). It is widely accepted that the LSFL-type LIPSSs arise from the interference of the incident laser radiation with a surface electromagnetic wave, generated at the roughly ablated surface^23,24^. For strong absorbing materials (such as metals and semiconductors) it has been demonstrated that the excitation of Surface Plasmon Polaritons (SPPs) plays a crucial role in the phenomenon^25,26^. The orientation is usually perpendicular to the laser beam polarization^27,28^ and the periods typically range between ~0.6λ and λ^29^.

The existence of HSFL allows for the generation of much smaller periods Λ down to ~λ/4–λ/5 in the case of diamond^30–32^. Indeed, HSFL with periodicities down to ~λ/8 can be found when graphitic-like phases are generated^33^. However, their origin is not fully understood and it is controversially debated in the literature^34–36^. In addition, the homogeneity of these structures and their formation still have not been effectively controlled, and therefore their use for the fabrication of high quality photonic devices is not considered in this work.

Controlling the aspect ratio *A* = *h/*Λ and morphology of the nanopatterns is crucial for photonic applications (being *h* the height of the nanostructures). Even though some studies suggest that a larger number of laser pulses *N* produces a proportional increase in *A*, a more detailed study of this phenomenon is still necessary^37^. Analogously, the spatial coherence, continuity and homogeneity of the nanopatterns over large areas is also relevant for real applications. While for LIPSSs nanopatterns in metallic surfaces achieving high spatial coherence is challenging^38^, for semiconductors this problem has been overcome by partially overlapping multiple laser pulses^12,39^.

In this work, the optimal conditions for the fabrication of high quality diamond nanostructures with customized photonic characteristics have been identified. A detailed study of the morphology and performance of the LIPSSs nanopatterns is presented by combining FEG-SEM and AFM analysis with FDTD simulations. The period Λ and the aspect ratio *A* of the nanopatterns were accurately adjusted by tuning the laser parameters, allowing to control their shape over arbitrarily large surfaces with a high degree of spatial coherence, homogeneity and reproducibility. The findings demonstrate a large potential for the production of high quality diamond photonic surfaces with tailored characteristics in a simple and reliable way.

## Modeling

In order to identify the potential of the LIPSSs nano-fabrication technique, FDTD simulation has been employed. In particular, the performance of moth-eye AR (Anti-Reflection) coatings fabricated via LIPSSs has been studied for a wide range of wavelengths in the near-IR.

Optical antireflection is traditionally accomplished employing single- or multiple-layer dielectric film coatings with certain required refractive indices and appropriate thickness profiles^40^. For diamond, which exhibits a high refraction index (around n ~ 2.4), suitable index-matched transparent materials are rare. In fact, for wavelengths in the near and mid-IR range, the necessary coating thickness scales up with the wavelength which results in stress buildup and thermal expansion mismatch (consequently lowering the coating adherence and damage threshold). Graded index surface relief structures have been also investigated in various wavelength ranges, but this technique usually involves complicated fabrication steps and imposes important challenges for device integration^41^.

Alternatively, AR coatings consisting on micro- and nanostructure arrays based on the moth-eye effect follow a different way of reducing reflectance^42^. In general, for structures with any dimensions of less than the irradiating wavelength, the sub-wavelength elements will no longer be distinguishable by the light and they will behave like propagating through an effective continuous medium. In this way, it becomes clear that for an appropriate range of wavelengths, the reflectance reducing physics of the AR structure is understood just exactly as for thin-film theory^43^. The extent on which this “homogenization” can be done has received plenty of attention in literature and different models have been proposed^44,45^. In this work, the Finite Difference Time Domain (FDTD) software LUMERICAL FDTD solutions v8.17, which is a nano-photonic Maxwell’s solver, was utilized. The transmission behavior for different spatial periods and aspect ratios of the LSFL patterns were thoroughly analyzed.

The first assumption made in the model was related to LSFL nanopatterns geometry. In general, the shape of the different surface structures has different gradient refractive index (GRIN) profile curves. This can be for example linear, parabolic, cubic, exponential, or sinusoidal, thus exhibiting different AR performance in terms of transparency and bandwidth. In particular, for diamond nanostructures fabricated via LIPSSs, the specific morphology and dimensions of the surface structures was accurately characterized^12^, where atomic force microscope profile traces show a good fit with parabolic shapes of the form *y*^2^ − 2*Rx* + (*K* + 1)*x*^2^ = 0. Here *R* is the radius of curvature and *K* is the conic constant (with K ≤ −1). The second assumption made was the maintenance of a spatial period/wavelength (Λ/λ) ratio such as only the zero^th^ diffractive orders propagate in the substrate and in the incident medium. Immediately from the grating equation, it can be deduced that this condition is matched when the rest of diffractive orders are evanescent:

1 $$\frac{{\rm{\Lambda }}}{\lambda } < \frac{1}{{\rm{\max }}({n}_{1},{n}_{2})+{n}_{1}(\sin \,\theta )}$$

where n_1_ and n_2_ are the refractive indexes of the surrounding medium and the substrate respectively, max holds for the maximum of the arguments and *θ* is the incident angle.

**Figure 2:**
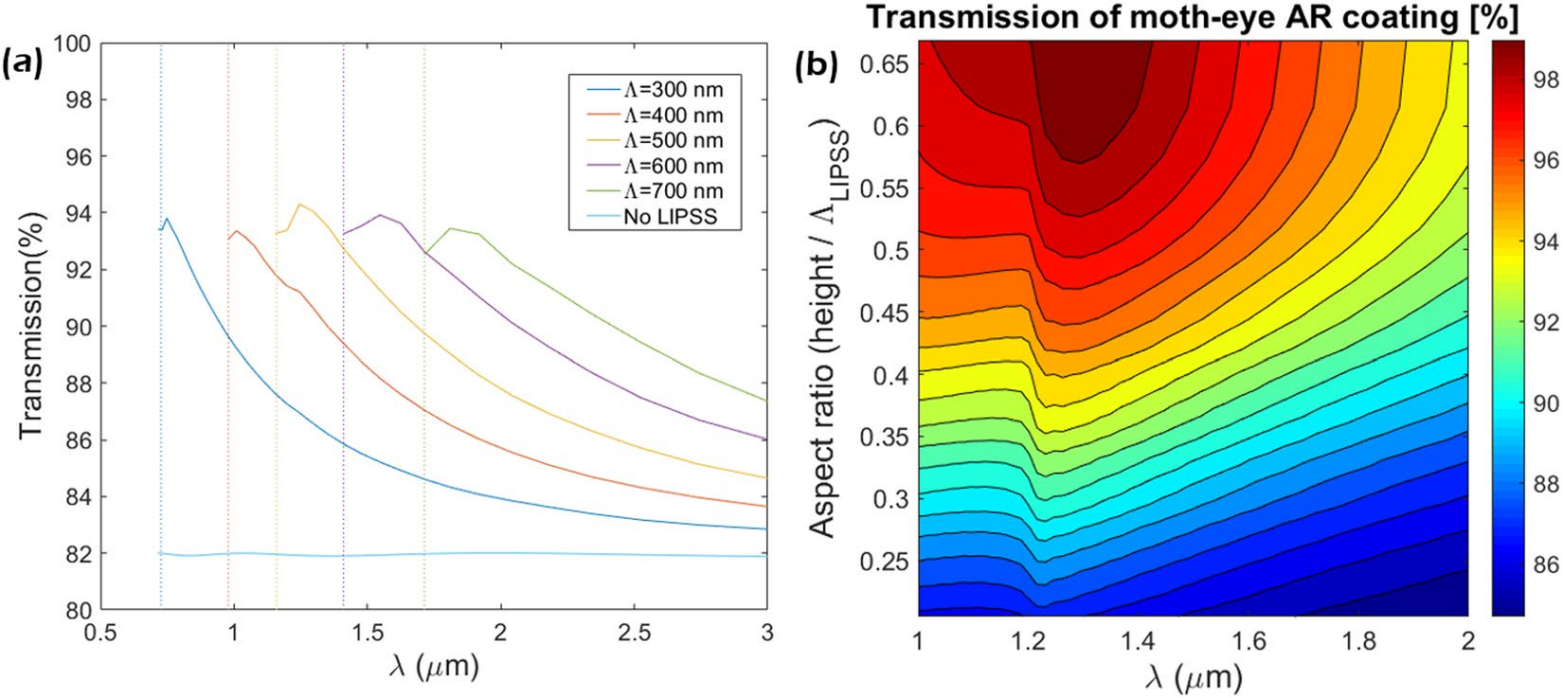
(**a**) Transmission of LIPSSs on diamond substrate as a function of LIPSSs spatial period (Λ) and incident wavelength with a fixed aspect ratio of 0.4. (**b**) Computed transmission of diamond LIPSSs as a function of aspect ratio and incident wavelength for Λ = 500 nm.

For example, a diamond nanostructure with periodicity Λ = 500 nm, will not have diffractive orders for λ > 1.21 μm. Figure 2(a) shows the transmission behavior for different LIPSSs periods Λ. Note that according to eq. (1), the range where no diffraction occurs changes with each period Λ, thus explaining the dashed lines picturing the below limit where diffraction ends. Otherwise, the results show that each Λ has its transmission peak located in different λ, which is explained by Bragg’s law. A similar maximum value of T ~ 94% for a common LIPSSs aspect ratio of *A* = 0.4 (compared to a T ~ 82% for a flat diamond surface). This reflectivity reduction can be further improved by scanning different values of the LIPSSs height, leading to a tunable different aspect ratio *A* = *h/*Λ as shown in Fig. 2(b). The transmission performance reached a maximum close to 100% for an *A* = 0.65.

The simulations highlight the importance of controlling the LIPSSs geometry in terms of Λ and *A* in order to tune the AR performance of the diamond surface. As mentioned in the introduction, the geometry of LSFL structures is highly controllable with the irradiation parameters, making them an excellent fabrication approach.

## Results and Discussion

### LIPSSs generation and geometry control

Diamond is a group IV semiconductor, and therefore laser-induced modification of the dielectric function transitorily changes the material properties to a metallic state^25,46^. Since the absorption of the femtosecond laser pulses by the diamond results in the generation of nearly free electrons in the conduction band on timescales shorter than the electron-phonon relaxation time, the response of the excited diamond is usually approximated by a free-carrier Drude response. During this plasmonically-active phase of the laser-irradiated diamond, the SPP-laser interference mechanism of inhomogeneous energy deposition is effective and leaves permanent imprints on the surface morphology after the conclusion of the pulse.

The spatial period Λ of LIPSSs nanopatterns under these conditions is known to depend on the laser wavelength, material plasmonic properties, the polarization of the laser electric field and the angle of incidence^47^. It is generally accepted that the period (Λ) of the LSFL is governed by the following equation:

2 $${\rm{\Lambda }}=\frac{\lambda }{(\eta +\,\sin \,\theta )}$$

where η = Re [(ε/(ε + 1)]^1/2^, is the real part of the effective refractive index of the air-diamond interface for surface plasmons, ε is the dielectric constant and θ the incidence angle of the laser light. By tuning the laser wavelength, is possible to control accurately the period Λ.

**Figure 3:**
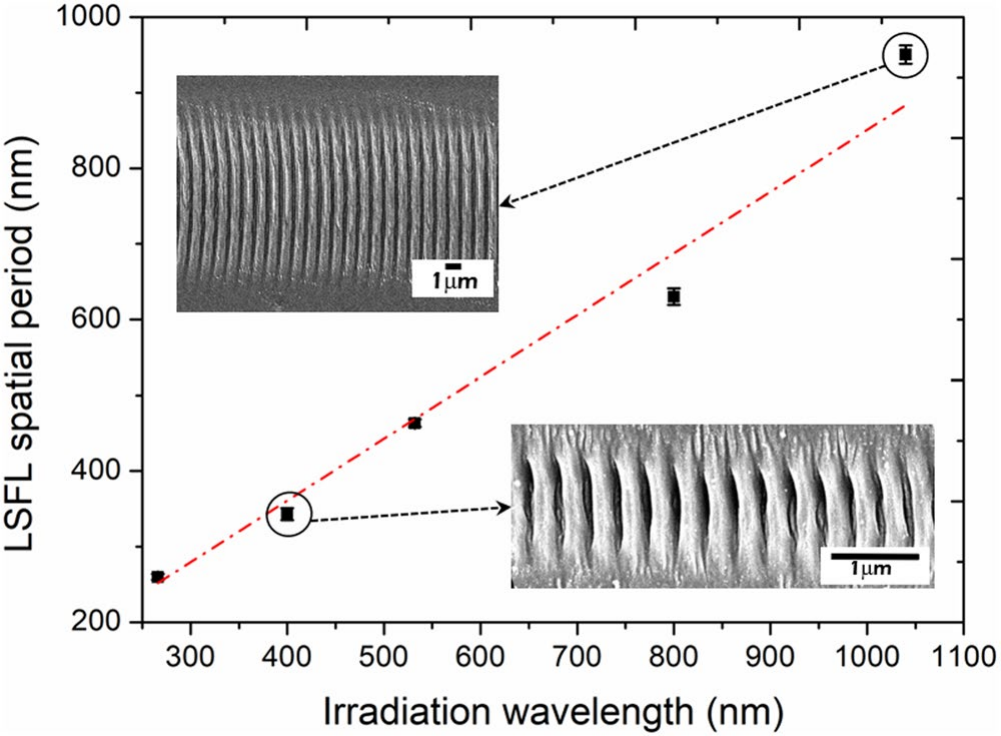
LSFL spatial period as a function of the irradiation wavelength. Insets: SEM images of the LSFL nanopatterns generated with λ = 1040 nm (above) and λ = 400 nm (below).

Experimentally, the samples were irradiated with five different wavelengths; firstly three different harmonics of a Ti:Sapphire laser, corresponding to IR light (λ ~ 800 nm), UV (λ ~ 400 nm) and deep-UV (λ ~ 267 nm), and also harmonics from an Ytterbium high repetition rate laser at a λ of ~520 nm and ~1040 nm. The pulse durations employed were 130 fs and 380 fs respectively. Figure 3 shows the obtained Λ as a function of the irradiation wavelength. The results showed a linear trend consisting of Λ ~ 0.85λ, which is in very good agreement with eq. (2), if the theoretical transient plasmon simulations values calculated for diamond are used^48^.

It is important to note that even though several works in the literature have suggested that Λ depends on *N*^29^ (an increase of *N* typically results in a decrease of Λ by several tens of percent^37^), this is not necessarily the case for diamond. Here, the spatial period of the generated LSFL structures remains fairly constant with the increase of *N*, at least up to ~70 pulses per spot which was the maximum value of *N* tested in this work. This result is consistent with a recent study reported by T. Apostolova *et al*.^48^, where they found that once surface ripples on diamond are formed, exposure by subsequent pulses has little effect on their spatial period. While other authors have reported a higher decrease of *Λ* with *N*, suggesting that interpulse feedback processes such as grating assisted effect play a role in this phenomenon^37^. These variations in the results suggest that the interpulse feedback processes may not have the same impact in diamond as in other materials as such as metals or semiconductors.

The majority of the fabricated LSFL nanopatterns were generated with an average of 20 pulses per spot and fluence values slightly above the ablation threshold. This value was found to be approximately 2 J/cm^2^, with the exception of the deep-UV irradiation case, where the ablation threshold was found to be approximately ~1 J/cm^2^, possibly caused by the multi-photon absorption coefficient of diamond at different wavelengths^5^. The fluence (F) value was varied from the ablation threshold to a maximum value of 6 J/cm^2^.

The morphology of the generated LSFL structures do vary within that range, and in order to produce high quality LSFL nanopatterns the total impinging cumulative fluence (*N* × F) should be in the order of 25–85 J/cm^2^. This result is significant since allows for a high degree of freedom in terms of laser irradiation parameters for producing high quality LSFL structures without an appreciable *Λ* variation.

### Analysis and control of the aspect ratio of nanostructures

**Figure 4:**
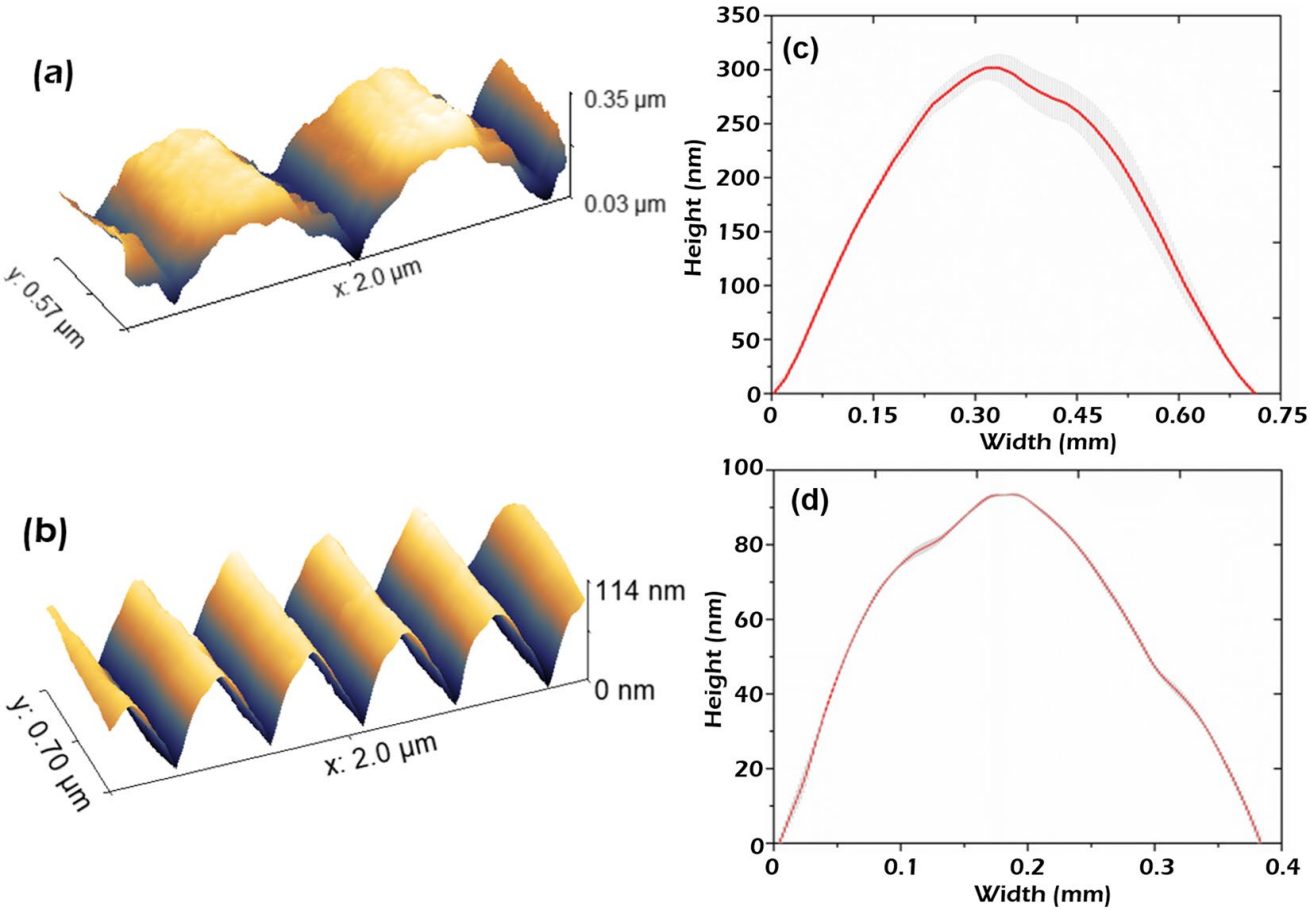
(**a**) AFM structure profile of an area irradiated with IR wavelength (λ ~ 800 nm) with 3.1 J/cm^2^ and 200 µm/s. (**b**) AFM structure profile of an area irradiated with UV wavelength (λ ~ 400 nm) with 4 J/cm^2^ and 200 µm/s. (**c**,**d**) Average profile with the standard deviation (gray shaded area) of the presented AFM scans.

The control of the aspect ratio *A* of nanostructures via modulation of the irradiation parameters is key for controlling the transmission behavior as shown in Fig. 2(b). In this section, to further characterize the geometry of the generated LSFL patterns, atomic force microscopy (AFM) was used to analyze the height profiles and surface roughness. As it can be seen in the AFM images and profiles presented in Fig. 4, the structures generated at 400 nm and 800 nm irradiation wavelengths present a very high degree of regularity and homogeneity. The height of the structures has been observed to depend on the irradiation wavelength as well as laser irradiation parameters, being the highest *A* ~ 0.45 obtained with 800 nm pulses.

**Figure 5:**
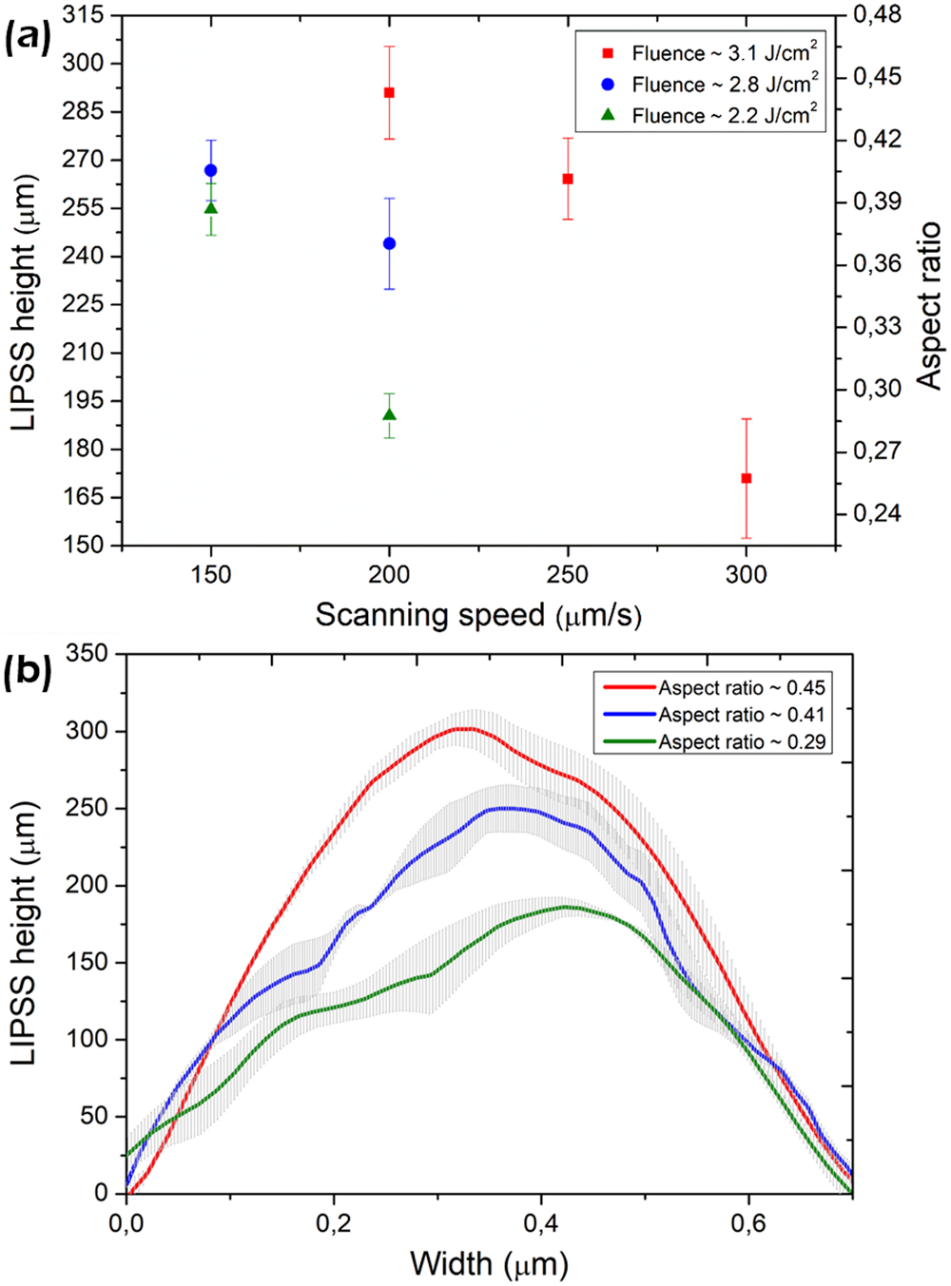
(**a**) AFM analysis of the LIPSSs height and aspect ratio *A* as function of fluence and scanning speed. (**b**) Average and standard deviation of structure profiles from fabricated nanopatterns with different *A* generated with: 3.12 J/cm^2^ and 200 µm/s (high *A*), 2.8 J/cm^2^ and 150 µm/s (intermediate *A*), 2.19 J/cm^2^ and 200 µm/s (low *A*).

In order to further investigate the effect of the laser parameters on *A*, AFM analysis were performed on the LSFL structures generated with 800 nm irradiation, at different fluences and scanning velocities. The results are presented in Fig. 5.

As it can be appreciated in Fig. 5(a), the *A* of the LFSL nanopatterns has a strong dependence on the laser irradiation parameters. As a common trend, once the LIPSSs threshold is reached, the aspect ratio depends fairly linearly with the number of pulses *N* employed, until the cumulative fluence is high enough for strong ablation to start. This result confirms that interpulse mechanisms are key to understand the variations on LIPSSs height. The applied fluence also plays a significant role, which main consequence is a higher absorption depth at higher pulse energies and consequently higher resulting *A*. Further studies are necessary to completely understand this behavior.

In Fig. 5(b), averaged AFM profiles are presented for different irradiation conditions, demonstrating the high degree of control achievable over the aspect ratio *A* of nanopatterns. The LSFL patterns with highest depth were obtained with a fluence of 3.12 J/cm^2^ and a scanning velocity of 200 µm/s (red line-out). Also, an intermediate profile (blue line-out) obtained with 2.8 J/cm^2^ and 150 μm/s is shown, together with a lower profile (green line-out) obtained with 2.19 J/cm^2^ and 200 μm/s.

### Nanopatterning with high spatial coherence and regularity over large areas

In order to extend the LIPSSs fabrication technique to large areas, it is important that the structures are spatially coherent and regular. The question of spatial coherence or phase distribution of the LIPSSs nanopatterns is also of importance for understanding the light-induced dynamics of LIPSSs formation in diamond.

To investigate the uniformity and coherence of the fabricated structures, a similar procedure to the one presented by Iaroslav Gnilitskyi *et al*.^38^ was followed: the local orientation of LIPSSs was analyzed on each SEM image, and from the distribution of the spatial orientation angle the spreading of the angle was extracted, which is commonly known as the dispersion of LIPSSs orientation angle (DLOA). According to this calculation, the average DLOA for the diamond patterns is 7.5° ± 2° for most irradiation conditions, indicating a high degree of regularity for all cases.

The physical mechanisms governing LIPSSs regularity have been already identified^38^, and it was linked with the decay length (i.e. the mean free path) of the excited surface electromagnetic waves (SEWs). The dispersion of the LIPSSs orientation angle correlates well with the SEWs decay length: the shorter this length, the more regular are the LIPSSs, which may explain the high regularity of LIPSSs patterns in diamond. In fact, using the calculated frequency dependence of the real and imaginary part of the inverse dielectric function of photoexcited carriers in diamond from^48^ (which is calculated for a similar intensity of 18 TW/cm^2^), and taking into account that the conduction electron density due to photo-ionization yields electron densities of around 10^22^ cm^−3^, it is customary to estimate that the SPP mean free path in diamond is well below 1 μm for the present experimental conditions, which is in accordance with our experimental observations regarding the LIPSSs regularity.

The model described in^48^, however, does not fully account for interpulse feedback processes. In general, the presence of a nanometric scale defect (either pre-existing in the surface or generated with the LIPSSs pattern) affects the subsequent structuration via localized surface plasmon (LSP) excitation around those isolated defects. The experimental data presented in this work, shows that once LSFL are formed, the spectrum of the surface roughness contains peaks at the SPP wavenumber causing enhanced inhomogeneous energy deposition, while the change on the LIPSSs aspect ratio is evidenced from the AFM measurements described in the previous section.

Furthermore, in this work it was observed that the LIPSSs patterns were automatically aligned with adjacent ones given that the spacing between them is sufficiently small, even though a few times larger than the spot size.

**Figure 6:**
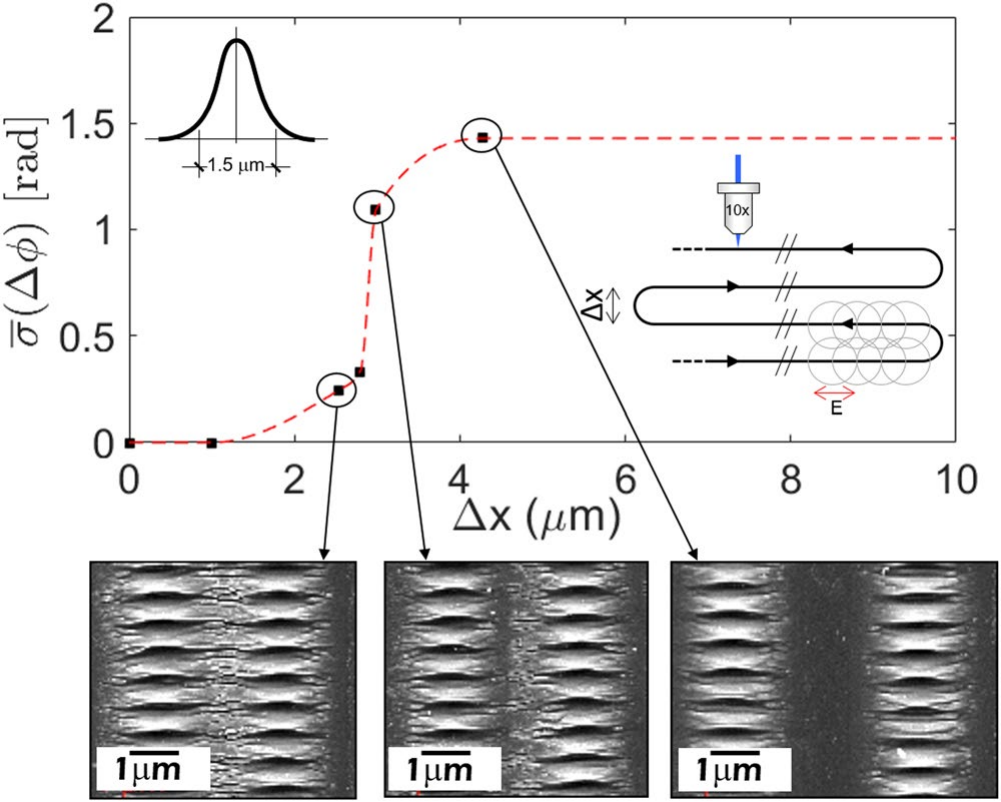
Standard deviation of the relative spatial phase between adjacent passes varying the distance for a 1.5 μm diameter spot. Highlighting the SEM images correspondent to adjacent passes separated approximately 2.5, 3 and 4 μm (from left to the right). Top inset: scheme to remark the laser spot diameter used in this study. Bottom inset: scheme of the scanning strategy followed in the fabrication process.

In order to further analyze the influence of the pre-existing surface morphology on the spatial phase of the LIPSSs pattern (spatial coherence between successive fabricated LIPSS nanopatterns), areas filled with structures perpendicular to the scanning direction (laser polarization collinear with the axis of movement) were produced, and varied the distance between adjacent passes (Δx), a scheme of the scanning procedure followed is presented in the bottom inset of Fig. 6.

Then, the spatial coherence of the different produced nanopatterned areas was analyzed in this work with a parameter that consisted on the averaged standard deviation of the relative spatial phase between adjacent passes. When this parameter is zero it means that complete spatial coherence has been achieved, while values of approximately 1.4 correspond to random alignment (i.e. no spatial coherence).

The results of this study are depicted in Fig. 6. As it is shown, the coherence in the LIPSSs pattern is relatively high for distances between adjacent passes of up to around Δx = 3 μm. Here, it is important to remark that this study was performed with a spot diameter of 1.5 μm (1/e^2^) as indicated in the top inset of Fig. 6. Therefore, the obtained results indicate that the spatial coherence is maintained for values of the distance between adjacent passes well beyond the spot radius or irradiated area (there is no overlap).

Although this study is not complete, it demonstrates that the LSP propagation reaches further than the laser irradiated area, suggesting that a coupling mechanism (possibly SPP mediated) with the adjacent nanostructures occurs.

**Figure 7:**
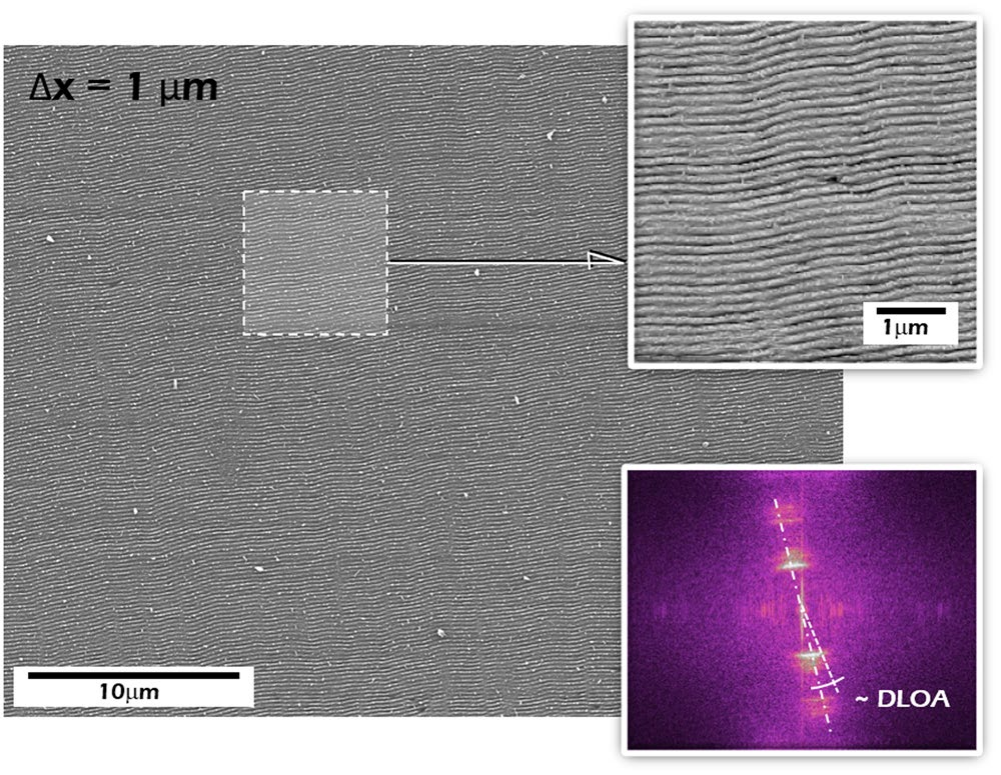
(**a**) SEM image of an area covered by highly coherent and homogeneous LSFL nanopatterns. Insets: Detailed view of the produced nanopatterns (above) and 2D-Fourier transform of the produced area, where the DLOA value is 7°.

Figure 7 shows SEM images of a large LIPSSs surface area fabricated with a distance between adjacent passes Δx = 1 μm, following the presented scanning approach (bottom inset Fig. 6) alongside with its 2D-FFT spectra and the DLOA.

## Conclusions

We have identified a convenient and simple methodology for tailoring the optical properties of diamond by employing laser induced periodic surface structuring (LIPSSs). The main laser parameters involved in the machining process have been identified, allowing for the control of the periodicity and height of the LIPSSs nanostructures with high accuracy. Nano-structuration in large areas with high quality and regularity has been demonstrated. While the present demonstrations have been centered in diamond anti-reflection (AR) coatings, the technique can be utilized in other semiconductor materials, extending the usability of the technique to many other areas of application.

## Materials and Methods

The samples consisted of single crystal Type IIa (Electronic grade, Element6) with [100] orientation diamond. The surfaces were cleaned using subsequent baths of boiling aqua regia and chromic acid to ensure clean surface before and after laser treatment.

The nanomachining process was performed in open air atmosphere with two different lasers. The first one was a Ti:Sapphire laser system consisting of a mode-locked oscillator and a regenerative amplifier. The system delivered up to 2 mJ, 130 fs pulses at a central wavelength of 800 nm (IR), with a 1 kHz repetition rate. The output of this laser was frequency doubled to 400 nm (UV) using a type I 500 μm thick BBO crystal, and then tripled to 267 nm using a type II 500 μm thick BBO crystal. The pulse energy was adjusted with a two-step setup: a variable attenuator formed by a half-wave plate and a low dispersion polarizer and neutral density filters. The UV and IR laser pulses were focused on the diamond samples utilizing a broadband 10x microscope objective with a NA of 0.16. The deep UV laser pulses were focused on the samples utilizing a 15x microscope objective with a NA of 0.32. The spot size on the samples was measured using a 50x microscope objective and a Coherent LaserCam HR-UV, yielding approximately a spot diameter of 1.5 µm (1/e^2^) at 400 nm and 5.5 μm at 800 nm.

The other laser setup used consisted of an Ytterbium solid state high repetition rate ultrafast laser with a pulse duration about 380 fs, which was able to be operated at a wavelength of *λ* = 1040 nm and *λ* = 520 nm. The laser source could be operated with variable pulse frequency up to 1 MHz. The maximum average power available behind the focusing optics was measured to be P = 3 W (at 1040 nm) and P = 1 W (working at 520 nm). The laser source was integrated in a laser processing machine (microSTRUCT vario, 3D-Micromac, Chemnitz, Germany). A galvanometer scanner was used to scan the laser beam across the sample together with a telecentric *f*-theta lens with a focal length of 100 mm. The spot size on the sample was measured yielding approximately a spot diameter of 10 µm at 1040 nm and 6 µm at 520 nm.

The characterization of the generated structures was performed with a JPK NanoWizard atomic force microscope in the intermittent contact mode using a silicon tip (r < 10 nm; aspect ratio <6: 1) with a force constant of 40 N m^−1^ and a resonant frequency of approximately 250 kHz. In addition to this, a 3D field-emission scanning electron microscope (FEG-SEM) system supplied by JEOL was used to obtain images of the LIPSSs nanopatterns. Free and open source software (Gwyddion) was used to analyze the AFM profiles and to obtain the two-dimensional Fast Fourier Transform (2D-FFT) of the different analyzed images. The Finite Difference Time Domain (FDTD) software LUMERICAL FDTD solutions v8.17 was used to characterize the transmission behavior of the LIPSSs nanostructures.
