# Author Correction: Tailoring diamond’s optical properties via direct femtosecond laser nanostructuring

**DOI:** 10.1038/s41598-018-36259-6

**Published:** 2018-11-27

**Authors:** M. Martínez-Calderon, J. J. Azkona, N. Casquero, A. Rodríguez, Matthias Domke, M. Gómez-Aranzadi, S. M. Olaizola, E. Granados

**Affiliations:** 1 CEIT-IK4 & Tecnun, Manuel Lardizabal 15, 20018 Donostia, San Sebastián Spain; 2 SLAC National Accelerator Laboratory, Menlo Park, CA 94025 USA; 3 Josef Ressel Center for Material Processing with Ultrashort Pulsed Lasers, Research Center for Microtechnology Vorarlberg University of Applied Sciences, Dornbirn, Austria

Correction to: *Scientific Reports* 10.1038/s41598-018-32520-0, published online 24 September 2018

This Article contains an error in the Y axis of Figure 5, where ‘LIPSS height (μm)’ should read ‘LIPSS height (nm)’. The correct Figure 5 appears below as Figure 1.

**Figure 1:**
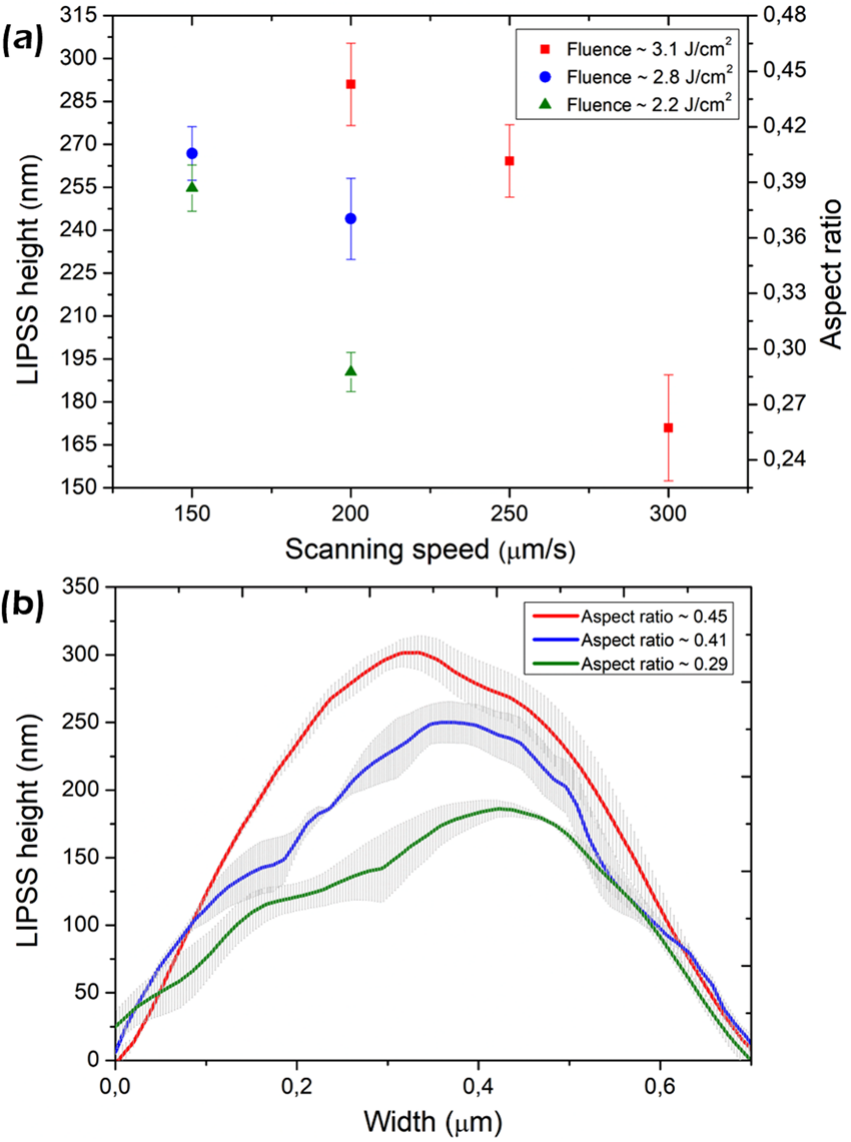
(**a**) AFM analysis of the LIPSSs height and aspect ratio *A* as function of fluence and scanning speed. (**b**) Average and standard deviation of structure profiles from fabricated nanopatterns with different *A* generated with: 3.12 J/cm^2^ and 200 μm/s (high *A*), 2.8 J/cm^2^ and 150 μm/s (intermediate *A*), 2.19 J/cm^2^ and 200 μm/s (low *A*).

